# Assisted clustering of gene expression data using ANCut

**DOI:** 10.1186/s12864-017-3990-1

**Published:** 2017-08-16

**Authors:** Sebastian J. Teran Hidalgo, Mengyun Wu, Shuangge Ma

**Affiliations:** 1 Department of Biostatistics, Yale University, 60 College Street, New Haven, 06520 USA; 2 School of Statistics and Management, Shanghai University of Finance and Economics, 777 Guoding Road, Shanghai, 200433 China; 3 Department of Statistics, Taiyuan University of Technology, 79 Yingze W St, Wanbailin Qu, Shanxi Sheng, 030024 Taiyuan Shi People’s Republic of China

**Keywords:** Assisted analysis, Clustering, Gene expression data

## Abstract

**Background:**

In biomedical research, gene expression profiling studies have been extensively conducted. The analysis of gene expression data has led to a deeper understanding of human genetics as well as practically useful models. Clustering analysis has been a critical component of gene expression data analysis and can reveal the (previously unknown) interconnections among genes. With the high dimensionality of gene expression data, many of the existing clustering methods and results are not as satisfactory. Intuitively, this is caused by “a lack of information”. In recent profiling studies, a prominent trend is to collect data on gene expressions as well as their regulators (copy number alteration, microRNA, methylation, etc.) on the same subjects, making it possible to borrow information from other types of omics measurements in gene expression analysis.

**Methods:**

In this study, an ANCut approach is developed, which is built on the regularized estimation and NCut techniques. An effective R code that implements this approach is developed.

**Results:**

Simulation shows that the proposed approach outperforms direct competitors. The analysis of TCGA (The Cancer Genome Atlas) data further demonstrates its satisfactory performance.

**Conclusions:**

We propose a more effective clustering analysis of gene expression data, with the assistance of information from regulators. It provides a new venue for analyzing gene expression data based on the assisted analysis strategy.

**Electronic supplementary material:**

The online version of this article (doi:10.1186/s12864-017-3990-1) contains supplementary material, which is available to authorized users.

## Background

In biomedical research, profiling studies have been extensively conducted. Information so collected has led to a better understanding of human genetics as well as practically useful models. In genetic research, gene expression data have been playing an essential role in the past decades. Compared to DNA and epigenetic changes, gene expressions are “closer” to phenotypes. With relatively mature techniques, they are also easy to measure at a genome-wide scale. Extensive methodological studies have been conducted on how to more effectively analyze gene expression data.

In the analysis of gene expression data, clustering has been playing an essential role. In some studies, clustering has been used to suggest/identify unknown functions of genes, with genes in the same cluster likely having related biological functions [1, 2]. In some other studies, clustering has been used as a way of reducing dimensionality. For example, some studies have suggested that conducting principal component analysis (and other analysis) on genes within the same clusters to reduce dimensionality is more sensible than doing that on all genes [3, 4]. In the literature, a large number of clustering methods have been developed and applied to gene expression data. Examples include the K-means, hierarchical clustering, agglomerative clustering, graph-based clustering, model-based sparse clustering, and others. For reviews and comprehensive discussions, we refer to [5, 6]. Although having certain technical differences, most of these methods share the common strategy of reducing “distance” within clusters while maximizing “distance” across clusters.

Despite great successes, it has also been suggested that clustering analysis of gene expression data still very often generates unsatisfactory results [7]. Gene expression studies usually have a limited sample size but a moderate to large number of genes. Intuitively, there is a lack of “sufficient information”. Multiple strategies have been developed to increase information. One strategy is to increase sample size via pooled analysis [8], which demands additional samples. There are also studies that use additional, especially biological, information [9]. In this study, we develop a different strategy which takes advantage of additional omics measurements on the same samples. Gene expression levels are regulated by multiple mechanisms, including copy number alteration, microRNA, methylation, and others. Intuitively, such regulators contain additional information on gene expressions. In recent biomedical research, a prominent trend is to conduct “multidimensional” profiling and collect data on gene expressions as well as their regulators on the same samples, making it possible to “borrow information” from other types of omics measurements for gene expression data analysis.

In this study, our goal is to conduct more effective clustering analysis of gene expression data. With the assistance of information from regulators. A novel clustering approach is developed to achieve this goal. The basic strategy is similar to that of minimizing distance within clusters and maximizing distance between clusters, and so the proposed approach has a solid statistical ground. Advancing from the existing methods, information of regulators is utilized in clustering gene expression data. More accurate clustering can be achieved by using more information. This is especially desirable with the decreasing cost of profiling but increasing cost of sample collection. This study differs from most of the existing ones that conduct the integrated analysis of multidimensional omics data. Studies such as [10, 11] aim at building more accurate disease outcome models by integrating gene expression and regulator data. Studies such as [12] aim at identifying “hot spots” of the chromosome that host multiple types of omics changes. In contrast, our goal is to conduct clustering, which is a more fundamental goal of gene expression data analysis. This study is warranted with an important analysis goal and an innovative and effective new method.

In what follows, we first describe the data structure. For the simplicity of notation, we use copy number alteration (CNA) as a representative of gene expression (GE) regulators. The proposed method will be directly applicable to other types of regulators. We then describe the proposed method, computational algorithm, and software development. Simulation and the analysis of TCGA (The Cancer Genome Atlas) data are conducted to gauge performance of the proposed approach relative to the alternatives. It is noted that although developed for GE data, the proposed approach can have broader applications.

## Methods

Consider a dataset with *n* iid samples. For the *i*th sample, assume that measurements are available on *p* GEs, denoted as *Y*_*i*_=(*Y*_*i*1_,*Y*_*i*2_,⋯,*Y*_*ip*_)^′^. In addition, assume that measurements are also available on *q* CNAs, denoted as *X*_*i*_=(*X*_*i*1_,*X*_*i*2_,⋯,*X*_*iq*_)^′^. For another type of regulator (for example, microRNA), the proposed approach is directly applicable. If there are two or more types of regulators, following [13], we can stack them together and create a “mega” vector of regulators. Our strategy is to use information in *X*_*i*_’s to assist the clustering of *Y*_*i*_’s. In the next subsection, we first describe our strategy for modeling the regulation between GEs and regulators. The assisted clustering method will then be developed.

### Modeling the GE-CNA regulation

Following the literature [14, 15], we describe the GE-CNA regulation using regression. Specifically, consider

$$Y_{i}=\boldsymbol{\beta}X_{i}+\epsilon_{i}, $$

where ***β*** is the matrix of unknown regression coefficients, and *ε*_*i*_ is the vector of “random errors”. In the literature, there are multiple ways of modeling the GE-CNA regulation. The regression approach has been adopted in quite a few recent studies and shown to have advantages over many alternatives for example the correlation-based. It is especially suitable for analysis with a large number of GEs and CNAs.

The regulation relationship is reflected in ***β***, with a nonzero component corresponding to a regulation between a GE and a CNA and the magnitude describing the strength of regulation. For estimating ***β***, we consider the penalized estimate

1 $${} \hat{\boldsymbol{\beta}}=\underset{\boldsymbol{\beta}}{\text{argmin}}\left\{ ||\boldsymbol{Y}-\boldsymbol{\beta}\boldsymbol{X}||_{2}^{2}+\lambda\left((1-\alpha)||\boldsymbol{\beta}||^{2}_{2} + \alpha||\boldsymbol{\beta}||_{1}\right)\right\},  $$

where ***Y*** and ***X*** are matrices consisting of *Y*_*i*_’s and *X*_*i*_’s, and *λ*>0 and 0≤*α*≤1 are data-dependent tuning parameters. The penalization approach is adopted to accommodate the high data dimensionality and for selection: for a specific gene, its expression level is expected to be affected by only a few CNAs, and a CNA is expected to affect the expression levels of only a few GEs, which poses a variable selection problem. The elastic net (Enet) penalty is adopted for its simplicity and to accommodate (possibly high) correlations among CNAs [16]. Similar estimation approaches have been adopted in the literature [17]. In data analysis, this estimation is effectively realized using the R package *glmnet*. The two tuning parameters *λ* and *α* are selected using V-fold cross validation (V=5 in our numerical study).

With the estimate $\hat {\boldsymbol {\beta }}$, denote the “predicted” GE values as $\hat {\boldsymbol {Y}}=\hat {\boldsymbol {\beta }} \boldsymbol {X}$, which describe the component of GEs regulated by the regulators. Accordingly, $\boldsymbol {Y}^{c}= \boldsymbol {Y} \setminus \hat {\boldsymbol {Y}}$ contains the levels of GEs regulated by other regulators (that are not included in ***X***), affected by other mechanisms, as well as “random variations”. This *decomposition* strategy for GEs has been recently developed in the literature [18] under other contexts and shown to provide important additional insights into GEs (beyond treating GEs as a whole). More discussions are also provided in the next section.

### Assisted clustering

For GEs, consider the weight matrix ***W***=(*w*_*jl*_)_*p*×*p*_, where the non-negative element *w*_*jl*_ measures the similarity between genes *j* and *l*. For a pair of the original GE measurements (as included in ***Y***), we define *w*_*jl*_ equal to the inverse of their Euclidean distance. Note that there are multiple ways of defining the similarity. This definition is adopted because of its simplicity. It shares a similar spirit with the popular K-means approach. Further, we define $\widehat {\boldsymbol {W}}$, which is obtained in a similar way as ***W*** but using $\hat {\boldsymbol {Y}}$, the regulated component of GEs.

Denote *A*_1_,…,*A*_*K*_ as a partition of {1,…,*p*} which leads to *K* disjoint clusters. For *A*_*k*_, denote $A_{k}^{c}$ as its complement. We propose the ANCut (Assisted NCut) measure as

2 $$ \text{ANCut}(A_{1},\ldots,A_{K})=\sum\limits_{k=1}^{K}\frac{\text{cut}\left(A_{k},A_{k}^{c};\boldsymbol{W}\right)} {\text{cutvol}\left(A_{k};\widehat{\boldsymbol{W}}\right)},  $$

where

3 $$ \text{cut}\left(A_{k},A_{k}^{c};\boldsymbol{W}\right)=\sum\limits_{j\in A_{k},l \in A_{k}^{c}} w_{jl},  $$

and

4 $$ \text{cutvol}\left(A_{k}; \widehat{\boldsymbol{W}}\right)=\sum\limits_{j,l \in A_{k}} \widehat{w}_{jl}.  $$

With a fixed *K*, the optimal clustering minimizes the ANCut measure.

**Rationale** The proposed approach has been motivated by the following considerations. It is built on the NCut technique, which is originally developed in imaging and other scientific fields [19] and more recently applied to genetic and other data types [20, 21]. The NCut technique may have multiple advantages over the alternatives. Specifically, the “cutting” step is relatively independent of the similarity/distance construction. Without making restrictive assumptions on the similarity measure and underlying data distributions and models, it enjoys very broad applicability. In addition, both the numerator and denominator have lucid interpretations, with the numerator measuring the across-cluster similarity and the denominator measuring the within-cluster similarity. More discussions on the NCut technique are provided in Additional file 1.

The most significant advancement from the existing approaches is that the numerator and denominator in (2) are defined using two different sets of similarity measures. The levels of GEs can be affected by multiple mechanisms. (a) One is regulating by the regulators measured in *X*. (b) Many (or most) existing studies are not “exhaustive” and do not measure all regulators. Thus, possibly there are regulators not included in *X*. (c) There are mechanisms other than regulation that may also affect GE levels. For example, the expression level of a gene can be affected by other genes, for example through RNA interference (which is also known as co-suppression or post-transcriptional gene silencing). Also, one gene can code for a transcription factor, and it can bind to the promoter region of another gene, which consequently affects its expression level. Following the literature [13, 18], in our analysis, we decompose GE levels into two components: the first is (a), and the second is (b)+(c).

Published studies [22, 23] under other contexts have shown that jointly considering the two components of GEs can lead to more sensible analysis results than considering them as a whole. Motivated by such observations, *our strategy is to minimize the NCut measures for both components of GEs*. Since the overall GE levels are equal to the sum of the two components, loosely speaking, using ***W*** (which measures the sum) and $\widehat {\boldsymbol {W}}$ is equivalent to using the two individual components. Our numerical analysis also confirms this intuition (results omitted). With this strategy, a seemingly more “straightforward” and “symmetric” objective function is $\sum \limits _{k=1}^{K}\frac {\text {cut}\left (A_{k},A_{k}^{c};\boldsymbol {W}\right)} {\text {cutvol}\left (A_{k};\widehat {\boldsymbol {W}}\right)}+\sum \limits _{k=1}^{K}\frac {\text {cut}\left (A_{k},A_{k}^{c};\widehat {\boldsymbol {W}}\right)} {\text {cutvol}\left (A_{k}; \boldsymbol {W}\right)}.$ Our exploration suggests that this objective function is computationally more expensive but leads to similar results as the one in (2).

**Fig. 1:**
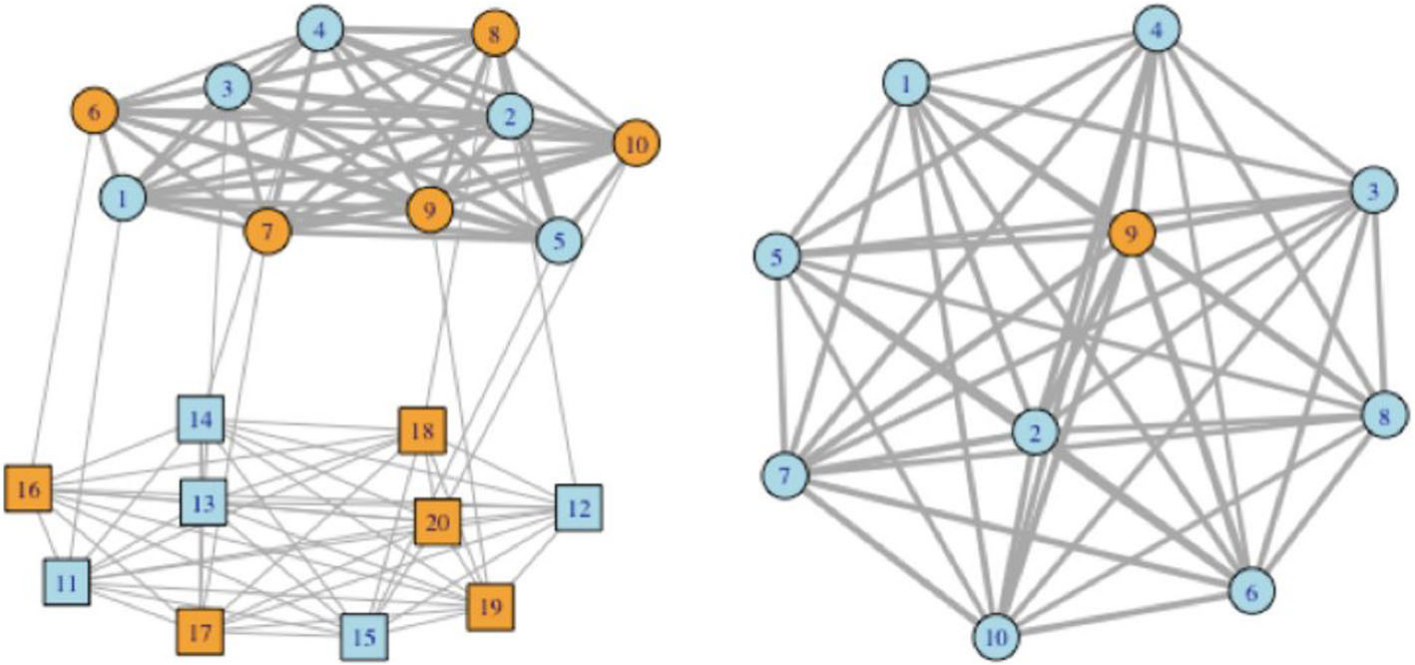
Toy example. A toy example with two clusters represented using different colors. *Circles* and *squares* represent GEs and CNAs, respectively. The *thickness of lines* represents the degree of similarity. *Left*: true structure as well as that recovered by the proposed approach. *Right*: K-means

**A toy example** To demonstrate the operating characteristics of the proposed method, we consider a toy example with 10 GEs and 10 CNAs. Data generation is the same as described in detail in the next section, except with a lower dimensionality. The true data structure is shown in the left panel of Fig. 1. There are a total of two equal-sized GE clusters, represented by different colors. The degree of similarity between two GEs (and CNAs) is represented by the thickness of lines. For the simulated dataset presented in Fig. 1, the proposed analysis is able to fully recover the true data structure with the penalized estimation and the true clustering structure with the ANCut approach. As an alternative, we also consider the popular K-means approach. We observe that the K-means approach completely fails for this dataset. It identifies two clusters one with nine GEs and the other with one GE. We have also experimented with some other alternatives and observe similar failing performance.

### Computation

For optimizing the objective function defined in (2), we adopt the simulated annealing (SA) technique [24]. Use *t* as the index of iteration. At iteration *t*, denote $A^{(t)}=\left \{A_{1}^{(t)},\dots,A_{K}^{(t)}\right \}$ as the partition (clustering result) and ANCut(*t*) as the value of the objective function. Further denote *B* as the maximum number of iterations. The value of *B* is not important, as long as it is large enough. Define the temperature function as *T*(*t*)=*L*log(*t*+1). In our numerical study, we set *L*=1000, which generates satisfactory results. In practice, to be prudent, other *L* values may also need to be examined. Extensive discussions on tuning parameter selection with the SA technique are available in the literature. The proposed algorithm proceeds as follows.

**Step 1** Randomly initialize $A^{(0)}=\left \{A_{1}^{(0)},\ldots,A_{K}^{(0)}\right \}$. In our numerical study, different initial values lead to almost identical results.

**Step 2** Set *t*=*t*+1. For *k*=1,…,*K*, compute *p*_*i*_ as the number of (*j*,*l*) pairs with $j, l \in A_{k}^{(t-1)}$. Draw *k*(−) and *k*(+) from {1,…,*K*} with probabilities proportional and inversely proportional to *p*_*i*_, respectively.

**Step 3** Draw *i* randomly from $A_{k(-)}^{(t)}$. Set $A_{k(+)}^{(t)}=A_{k(+)}^{(t-1)}\cup \{i\}$ and $A_{k(-)}^{(t)}=A_{k(-)}^{(t-1)}\setminus \{i\}$. For *j*≠*k*(+),*k*(−), $A_{j}^{(t)}:=A_{j}^{(t-1)}$.

**Step 4** If ANCut(*t*)≤ANCut(*t*−1), keep *A* ^(*t*)^ as it is. If not, keep *A* ^(*t*)^ as it is with probability $\exp \Bigg (-\frac {\text {ANCut} (t)- \text {ANCut} (t-1)}{T(t)} \Bigg)$, and otherwise *A* ^(*t*)^=*A* ^(*t*−1)^.

**Step 5** Repeat Steps 2-4 until *t*=*B*.

Extensive research on the SA technique is available in the literature [24]. In Step 2, the proposed probabilities prefer adding a new member to a small cluster and deleting a member from a large cluster. Thus, the “prior” is that clusters have similar sizes. Note that this strategy may be somewhat subjective and can be adjusted according to the specific scientific context. Convergence of the SA algorithm to the global optimizer has been established in the literature [24]. It is achieved in all of our numerical examples.

The proposed analysis, which consists of penalized estimation and ANCut, is computationally affordable. The two steps have computational complexity *O*(*n* *p* *q*) and *O*(*B* *p*), respectively. For a simulated dataset with *p*=*q*=200 and *n*=50, we consider 100 tuning parameter values in penalized estimation and *B*=10,000 in ANCut. The proposed analysis takes about 30 s on a laptop with standard configurations.

**Software** To facilitate data analysis, we develop an R package *NCutYX* and make it publicly available at https://github.com/shuanggema. If the R library *devtools* is installed, then the *NCutYX* package can be easily installed using devtools::install_github(“shuanggema/NCutYX”). The proposed approach is implemented using the function *ANCut*, which proceeds as follows: clust ←ANCut(Y, X, K = 2, B = 3000, L = 1000, alpha = 0.5, nlambdas = 100, ncv = 5, dist = “euclidean”) In the above command, *Y* is the data matrix of GEs, *X* is the data matrix of regulators, *K* is the number of clusters, *B* is the number of SA iterations, *L* is the temperature coefficient, alpha is *α* in the Enet penalty (note that in this sample command, we fix *α* as its default value. It is easy to data-dependently select *α*), nlambdas is the number of *λ* values in Enet, ncv is the number of cross-validations, and dist specifies that the Euclidean distance is used in defining the dissimilarity. The resulting object *clust* is a list where the first entry (*clust[[1]]*) is a vector of SA sequence, the second entry (*clust[[2]]*) includes the clustering results, and the third entry (*clust[[3]]*) contains the optimal *λ* value. Written in a friendly way, the package can be easily adopted and modified.

## Results

### Simulation

Use the same notations as in the last section. In simulation, GEs and CNAs are linked with the regression model ***Y***=***β*** ***X***+***ε***. Each element of the matrix ***ε***, denoted by *ε*_*i*,*j*_, is iid from a normal distribution with mean=0 and sd=2. For each subject, the CNA values are generated from a multivariate normal distribution with marginal means 0 and variance-covariance matrix ***Σ***. ***Σ*** has a block-diagonal structure with two blocks, each of which has size *q*/2. In each block, the diagonal elements are equal to 1, and all off-diagonal elements are equal to *ρ*. In TCGA (which is analyzed in the next section) and other datasets, it has been observed that the processed CNA data have unimodal distributions close to normal (although the raw data may have different distributional characteristics). Two equal-sized CNA clusters are generated, with those in the same cluster correlated and different clusters uncorrelated. For the regression coefficient matrix, consider

$${} {{\boldsymbol{\beta}=\left[\!\begin{array}{cccccccc} \beta_{11} & \beta_{12} & \cdots & \beta_{1\ q/2} & 0 & 0 &\cdots & 0\\ \beta_{21}&\beta_{22} & \cdots & \beta_{2\ q/2} & 0 & 0 &\cdots & 0 \\ \vdots & &\ddots &\vdots & \vdots &\vdots & \vdots & \vdots \\ \beta_{p/2\ 1}& \beta_{p/2\ q/2}&\cdots & \beta_{p/2\ q/2} & 0 & 0 &\cdots & 0\\ 0&0 & \cdots & 0 & \beta_{p/2+1\ q/2+1} & \beta_{p/2+1\ q/2+2} & \cdots & \beta_{p/2+1\ q}\\ 0&0 & \cdots & 0 & \beta_{p/2+2\ q/2+1} & \beta_{p/2+2\ q/2+2} & \cdots & \beta_{p/2+2\ q}\\ \vdots & &\ddots &\vdots & \vdots & \vdots &\vdots & \vdots\\ 0&0 & \cdots & 0 & \beta_{p\ q/2+1} & \beta_{p\ q/2+2} & \cdots & \beta_{p\ q}\\ \end{array}\!\right].}} $$

For each column, with the sparsity consideration, we set *q*_0_ coefficients to be nonzero and the rest zero. We consider two sparsity levels with *q*_0_= 3 and 6. Two different coefficient settings are considered. The first (C1) has the nonzero coefficients randomly generated from Uniform [*h*/2,*h*], where the parameter *h* determines the strength of regulation. The second (C2) has the nonzero coefficients randomly generated from Uniform [−0.15,0.25], which describes the scenario where CNAs have both positive and negative regulations on GEs and the amount of positive and negative regulation is not equal. Under this data generating structure, there are two clusters of CNAs/GEs, and GEs in the first (second) cluster are regulated by CNAs in the first (second) cluster. Beyond sparsity, this setting also describes the “localization” of regulations.

Under this simulation setting, the component of GE that is regulated by regulators, i.e., effect “(a)” in the “Assisted clustering” section, is ***β*** ***X***. For the other component of GE, i.e., effects “(b)+(c)”, is randomly generated. There are at least two considerations for this. First, unlike for the regulators’ effects, research on the second component of GE is still much limited. It is not entirely clear how to simulate effects “(b)+(c)”. More importantly, this setting favors the alternative approaches (to be described below). If the proposed approach has competitive performance under this unfavorable setting, it is reasonable to expect better results under favorable settings.

When evaluating performance of the proposed approach, we consider both accuracy and stability. With a set of clusters {*A*_1_,…,*A*_*K*_}, an adjacency matrix ***A***=(*a*_*jl*_)_*p*×*p*_ can be constructed, where the (*j*,*l*)th element *a*_*jl*_=1 if *j*,*l*∈*A*_*k*_,*k*=1,⋯,*K* and 0 otherwise. Let ***A***_*T*_ and $\hat {\boldsymbol {A}}$ be the adjacency matrices of the true and estimated clusters, respectively. Then the accuracy measure is defined as the diversity between ***A***_*T*_ and $\hat {\boldsymbol {A}}$,

5 $$ M_{accuracy}=\sum_{j,l}(\boldsymbol{A}_{T} \odot \hat{\boldsymbol{A}})_{jl}/p^{2},  $$

where ⊙ is the component-wise product. With *N* replicates, denote $\widehat {\boldsymbol {A}}^{(1)},\ldots,\widehat {\boldsymbol {A}}^{(N)}$ as the adjacency matrices as constructed above. We define the stability measure as

6 $$ M_{stability}={N \choose 2}^{-1}\sum_{1\leq N_{1}<N_{2}\leq N}\sum_{j,l}|\widehat{\boldsymbol{A}}^{(N_{1})}-\widehat{\boldsymbol{A}}^{(N_{2})}|_{jl}/p^{2}  $$

This definition shares a similar spirit with the U-statistic stability measure [25]. For *M*_*accuracy*_, a smaller value suggests a higher accuracy; For *M*_*stability*_, a smaller value suggests more stable results.

#### A closer look at the proposed approach

**Fig. 2:**
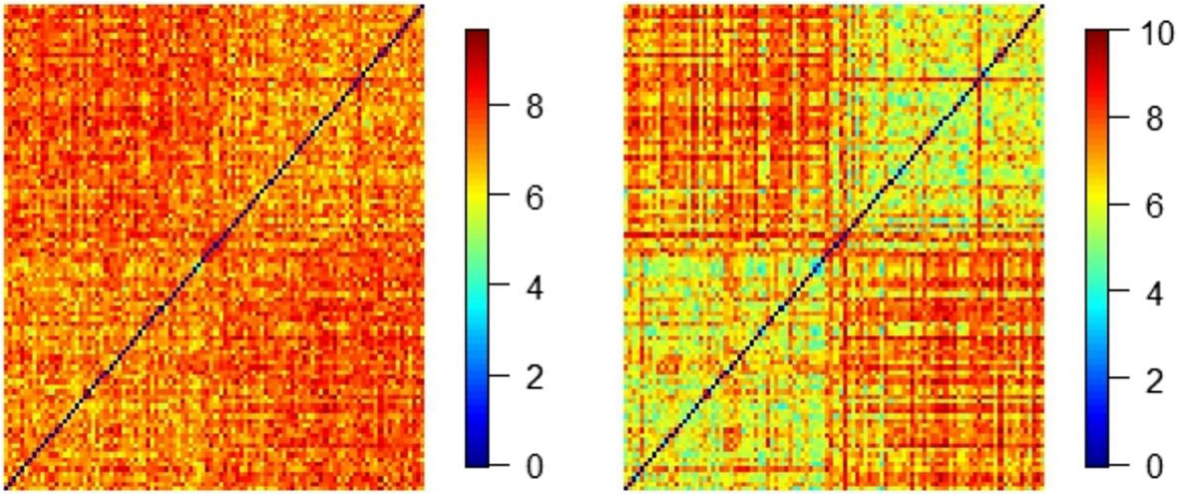
Heatmap of simulated GEs. Heatmaps of GEs for one simulated replicate. *Left*: using the observed GE values. *Right*: using the predicted GE values, where the two-cluster structure is more clearly seen

Consider the setting with coefficient C1, with *p*=100, and *q*=*p*/2. In addition, set *ρ*=0.10 and *h*=0.10. We first simulate one replicate and present the heatmaps for the observed and predicted GE levels in Fig. 2. It is observed that the heatmap using the predictive GEs shows a clearer clustering structure. This provides an intuitive justification for the proposed strategy of using CNA information.

Consider the true clustering structure (*A*_1_,*A*_2_). Further consider $\text {NCut}(A_{1}, A_{2})=\sum \limits _{k=1}^{2}\frac {\text {cut}\left (A_{k},A_{k}^{c};\boldsymbol {W}\right)} {\text {cutvol}\left (A_{k};\boldsymbol {W}\right)}$, which is the objective function defined in (2) using the observed GEs for both numerator and denominator, and ANCut(*A*_1_,*A*_2_). With 100 simulated replicates, the mean values are

7 $$ \text{Ncut}(A_{1},A_{2})=1.67 \quad \text{and} \quad \text{ANcut}(A_{1},A_{2})=1.46.  $$

This suggests that the proposed assisted analysis can more strongly define the clustering structure.

We further examine the potential gain by using information of CNAs for clustering GEs. Specifically, beyond the proposed ANCut, we also consider the popular K-means approach. As the simulated GEs have normal distributions, K-means is favored. In addition, we also consider the approach ANCut_*T*_, which is the proposed ANCut but with ***β*** in the place of $\hat {\boldsymbol {\beta }}$. Under this approach, we have perfect information on the GE-CNA regulation. As shown in Table 1, multiple scenarios on *n*, *p*, and *h* are considered. With 100 replicates, we compute the mean accuracy measure *M*_*accuracy*_. It is observed that if the regulation is perfectly known, then using ANCut leads to perfect identification of clusters. If the regulation needs to be estimated, using the proposed ANCut leads to accuracy better than or similar to that of K-means. This further justifies the value of using regulator information.

**Table 1:** Simulation: mean *M*_*accuracy*_ measures over 100 replicates

*n*	*p*	*h*	ANCut_*T*_	ANCut	K-means	*n*	*p*	*h*	ANCut_*T*_	ANCut	K-means
100	100	0.10	0%	17%	27.8%	100	100	0.20	0%	3.6%	2.5%
100	150	0.10	0%	6.5%	7.2%	100	150	0.20	0%	0.2%	0.1%
100	200	0.10	0%	1.9%	1.6%	100	200	0.20	0%	0.05%	0.01%
150	100	0.10	0%	11.5%	20.9%	150	100	0.20	0%	1.3%	1.1%
150	150	0.10	0%	3%	3.2%	150	150	0.20	0%	0.02%	0.02%
150	200	0.10	0%	0.4%	0.4%	150	200	0.20	0%	0.01%	0%
200	100	0.10	0%	8.3%	14.3%	200	100	0.20	0%	0.06%	0.08%
200	150	0.10	0%	1.6%	1.8%	200	150	0.20	0%	0.01%	0.01%
200	200	0.10	0%	0.02%	0.02%	200	200	0.20	0%	0%	0%

#### Comparison with the alternative methods

To better gauge performance of the proposed method, we compare with competing alternatives including K-means and spectral clustering (Spec.) [26]. These two alternatives are considered because of their popularity and satisfactory performance observed in published studies [27–29]. We also consider NCut to better appreciate the merit of assisted analysis. We comprehensively consider multiple combinations of *n*,*p*,*q*,*q*_0_,*h*, and *ρ* values for coefficient setting C1. We also examine multiple scenarios under coefficient setting C2 with various *n*,*p*,*q*,*q*_0_, and *ρ*=0.40. There are a total of 80 simulation settings. Under each setting, we simulate 100 replicates. Summary statistics are presented in Tables 2, 3, 4, 5, and 6. Performance of the proposed approach depends on the strength of GE-CNA regulation, correlation of CNAs, data dimensionality, sample size, and others, in a similar way as observed for other clustering methods. Across the whole range of simulation settings, the proposed method is observed to have competitive performance, with superior accuracy and stability. For example, in Table 2 with *n*=200, *p*=500, *q*=500, and *q*_0_=6, the proposed method has *M*_*accuracy*_ equal to 28.4%, compared to 42.4% (NCut), 41.8% (K-means), and 37.4% (Spec.). It also has satisfactory performance in terms of stability. Under this specific setting, the *M*_*stability*_ values are 17.6% (proposed), 40.6% (NCut), 42.2% (K-means) and 34.5% (Spec.). It is observed that performance of the proposed method decays with the increase of dimensionality and correlation among CNAs. This observation is reasonable as the proposed method involves estimating the regulation relationship. The matrix ***β*** has a total of *q*×*p* parameters, which can be very difficult to estimate with a moderate sample size. This estimation gets challenged with an increase in data dimensionality and correlation. When CNAs have both positive and negative regulation effects on GEs, of which the results are provided in Table 6, the proposed method also performs better than the alternatives. For example, with *n*=400, *p*=500, *q*=250, and *q*_0_=6, the proposed method has *M*_*accuracy*_ equal to 33.6%, compared to 34.5% (NCut), 39.4% (K-means) and 40.4% (Spec.). It also has satisfactory performance in terms of stability. Under this specific setting, the *M*_*stability*_ values are 15.2% (proposed), 19.3% (NCut), 17.3% (K-means) and 16.2% (Spec.).

**Table 2:** Simulation under coefficient setting C1 with *h*=0.15 and *ρ*=0.20: mean values based on 100 replicates

Parameters				*M*_*accuracy*_				*M*_*stability*_			
*n*	*p*	*q*	*q*_0_	ANCut	NCut	K-means	Spec.	ANCut	NCut	K-means	Spec.
200	500	250	3	44.8%	49.8%	49.8%	49.7%	48%	48.3%	49.5%	42.5%
200	500	250	6	27.7%	41.8%	41.9%	36.8%	46.2%	47.1%	49.3%	33.8%
200	500	500	3	45%	49.7%	49.8%	49.7%	28.4%	44.3%	47.7%	42.8%
200	500	500	6	28.4%	42.4%	41.8%	37.4%	17.6%	40.6%	42.2%	34.5%
400	500	250	3	38.2%	49.5%	49.7%	49.5%	47.7%	45.2%	49.6%	43.5%
400	500	250	6	16.8%	25%	25.5%	23.9%	41.6%	40.7%	49%	24%
400	500	500	3	38.4%	40.4%	49.7%	49.5%	25.3%	30.1%	48.1%	43.3%
400	500	500	6	16.8%	23.7%	25.2%	24.5%	12.4%	25.7%	24.4%	24.3%
200	800	400	3	45.2%	49.8%	49.8%	49.7%	48.4%	46%	49.8%	43.3%
200	800	400	6	18.2%	33.3%	20.4%	33.9%	42.9%	33.7%	47.3%	30.1%
200	800	800	3	45.4%	49.7%	49.8%	49.7%	32.8%	48.2%	48.1%	42.4%
200	800	800	6	29.3%	36.5%	34.2%	33.7%	22.8%	36.6%	29.6%	30%
400	800	400	3	39.6%	49.7%	49.6%	48.8%	47.8%	48.1%	49.7%	44.1%
400	800	400	6	29.4%	22.6%	34.1%	20.3%	46.7%	23.1%	49.2%	20.3%
400	800	800	3	39.5%	49.8%	49.4%	48.7%	28.8%	48.9%	48.4%	44.2%
400	800	800	6	27.9%	22.6%	34.2%	21.1%	19.7%	19.4%	24.5%	18.3%

**Table 3:** Simulation under coefficient setting C1 with *h*=0.15 and *ρ*=0.40: mean values based on 100 replicates

Parameters				*M*_*accuracy*_				*M*_*stability*_					
*n*	*p*	*q*	*q*_0_	ANCut	NCut	K-means	Spec.	Aug-K	ANCut	NCut	K-means	Spec.	Aug-K
200	500	250	3	40.6%	49.1%	49.3%	48.3%	99.7%	47.6%	48.1%	49.5%	41.7%	1%
200	500	250	6	17.4%	20.8%	20.9%	20.6%	46.1%	42.3%	36.1%	47.4%	20.7%	21.6%
200	500	500	3	40.7%	49.6%	49.1%	48.5%	99.5%	29.4%	48.3%	47.2%	42.6%	1%
200	500	500	6	17.2%	20.3%	20.3%	20.6%	58.3%	12.5%	14.8%	15.8%	20.6%	22.3%
400	500	250	3	30%	48.1%	47.5%	43.1%	99.9%	46.7%	47.8%	49.6%	40.2%	0.1%
400	500	250	6	7.4%	19.6%	9%	9%	24.9%	33.6%	34.7%	39.4%	11.5%	20.8%
400	500	500	3	30.8%	48.5%	47.7%	43.6%	99.9%	20.9%	37.5%	47.4%	39.9%	0.5%
400	500	500	6	7.6%	9%	9.4%	9.4%	30.7%	6.9%	6.7%	7.7%	11.8%	23%
200	800	400	3	41%	49.7%	48.2%	45.8%	99.7%	47.9%	49.5%	49.5%	41.2%	0.4%
200	800	400	6	18.5%	20.9%	18.8%	18.7%	52%	43.2%	22.5%	44.7%	18.6%	20.7%
200	800	800	3	41.4%	49.3%	48.4%	45.8%	99.6%	32.6%	46.8%	47.1%	40.5%	1%
200	800	800	6	18.6%	18.4%	19%	18.6%	62%	13.4%	11.9%	16.4%	18.8%	20%
400	800	400	3	31.5%	49.7%	42%	37.1%	99.9%	47%	46.8%	49.6%	33.9%	0.1%
400	800	400	6	9.4%	6.9%	7.9%	8%	27.4%	35.2%	14.5%	35.6%	10.3%	18.7%
400	800	800	3	32.5%	49.4%	43%	39.1%	99.9%	11.7%	43.6%	7.7%	10.2%	0.1%
400	800	800	6	9.3%	7.6%	8%	8.2%	31.3%	10.9%	4.1%	6.6%	10.2%	9.1%

**Table 4:** Simulation under coefficient setting C1 with *h*=0.25 and *ρ*=0.20: mean values based on 100 replicates

Parameters				*M*_*accuracy*_				*M*_*stability*_			
*n*	*p*	*q*	*q*_0_	ANCut	NCut	K-Means	Spec.	ANCut	NCut	K-Means	Spec.
200	500	250	3	34%	47.5%	47.6%	45.1%	47.2%	45.4%	49.5%	40.3%
200	500	250	6	11.8%	13%	13.7%	13.6%	39.1%	14.1%	43%	15.6%
200	500	500	3	33.9%	47.5%	47.6%	44.9%	21.2%	38.5%	46.5%	39.9%
200	500	500	6	12.3%	14.2%	14.2%	14%	9.9%	11.8%	10.6%	15.2%
400	500	250	3	24%	38.5%	40.5%	34.6%	44.5%	37.4%	49.6%	32.3%
400	500	250	6	4.4%	4.6%	4.8%	4.8%	28%	15.3%	31.3%	7.1%
400	500	500	3	23.8%	37.8%	40.6%	34.7%	15.4%	30.3%	40.9%	32.3%
400	500	500	6	4.4%	4.7%	5%	5.1%	4.6%	12.1%	4.5%	7.3%
200	800	400	3	35%	45.7%	43.8%	39.6%	47.4%	43.4%	49.5%	34.8%
200	800	400	6	14.2%	12.9%	12.8%	12.8%	40.3%	43.1%	39.9%	14.5%
200	800	800	3	35.3%	45.6%	44.2%	40.3%	27%	43.1%	42.9%	35.3%
200	800	800	6	14.4%	13.8%	13.1%	12.8%	15%	12%	9.7%	14.3%
400	800	400	3	25.3%	44.4%	30.8%	29.3%	45.4%	43.1%	49.3%	26.1%
400	800	400	6	6.6%	3.6%	4.6%	4.3%	30.7%	14.9%	29.8%	6.5%
400	800	800	3	25.3%	32.1%	30.9%	29.4%	19.6%	21.1%	25.5%	26.2%
400	800	800	6	6.5%	3.9%	4.4%	4.6%	8.6%	8.4%	3.9%	6.5%

**Table 5:** Simulation under setting coefficient C1 with *h*=0.25 and *ρ*=0.40: mean values based on 100 replicates

Parameters				*M*_*accuracy*_				*M*_*stability*_			
*n*	*p*	*q*	*q*_0_	ANCut	NCut	K-means	Spec.	ANCut	NCut	K-means	Spec.
200	500	250	3	24.8%	29.8%	32%	29.5%	45.1%	44.8%	49%	26.7%
200	500	250	6	4.5%	4.6%	5%	4.6%	29.6%	21.3%	30.1%	6.5%
200	500	500	3	25.6%	28.3%	31.8%	29.8%	15.9%	17.7%	27.5%	26.7%
200	500	500	6	4.4%	4.5%	5%	4.7%	4.2%	6.8%	4.8%	6.6%
400	500	250	3	14.3%	16.5%	18.3%	17.3%	38.9%	43.5%	46.5%	17.4%
400	500	250	6	1%	1.1%	1.1%	1.1%	16%	11.5%	16.8%	1.9%
400	500	500	3	14.9%	20.4%	18.7%	17.7%	10.6%	23.1%	14.2%	17.8%
400	500	500	6	1%	1.3%	1.3%	1%	1.1%	1.3%	1.8%	1.9%
200	800	400	3	25.8%	28.2%	27.1%	26.5%	45.7%	43.4%	47.8%	23.7%
200	800	400	6	7.3%	4.9%	4.3%	4%	30.6%	16.7%	28.9%	6.1%
200	800	800	3	26.2%	26.5%	27.7%	26.8%	20.3%	23%	19.6%	23.7%
200	800	800	6	7.1%	4.4%	4%	3.9%	25.4%	10.6%	20.1%	5.1%
400	800	400	3	16.1%	16.4%	15.9%	15.7%	40.5%	31.5%	42.6%	16%
400	800	400	6	4.6%	1.2%	1%	0.9%	19%	5%	15.4%	1.7%
400	800	800	3	16.2%	17.4%	15.9%	16%	14.7%	14.1%	11.2%	15.8%
400	800	800	6	3.6%	1%	0.9%	1%	5.8%	5.4%	0.9%	1.7%

**Table 6:** Simulation under coefficient setting C2 with *ρ*=0.40: mean values based on 100 replicates

Parameters				*M*_*accuracy*_				*M*_*stability*_			
*n*	*p*	*q*	*q*_0_	ANCut	NCut	K-means	Spec.	ANCut	NCut	K-means	Spec.
200	500	250	3	49.9%	49.9%	50%	49.9%	31.2%	32.6%	47.9%	33.7%
200	500	250	6	41.6%	42.4%	48.5%	46.2%	24.1%	27.1%	46.3%	37.9%
200	500	500	3	49.2%	49.5%	50%	50%	31.2%	32.1%	47.9%	33.7%
200	500	500	6	41.9%	43.9%	48.7%	44.1%	24.3%	20.1%	46.6%	17.9%
400	500	250	3	46.3%	47.6%	49.9%	48.8%	28.9%	30.1%	47.9%	41.6%
400	500	250	6	33.6%	34.5%	39.4%	40.4%	15.2%	19.3%	17.9%	16.2%
400	500	500	3	46.2%	48%	49.8%	47.1%	28.3%	31.3%	47.9%	31.4%
400	500	500	6	34.2%	34%	35.3%	33.9%	16.5%	17.4%	19.6%	14.1%
200	800	400	3	48.6%	49.3%	49.9%	49.3%	32.8%	35.1%	47.6%	45.9%
200	800	400	6	42.3%	43.3%	46.3%	44.3%	29.3%	30.1%	41.8%	33.8%
200	800	800	3	48.6%	49%	49.9%	48.1%	32.5%	36.7%	47.9%	29.4%
200	800	800	6	42.1%	39.3%	46.5%	43.9%	29.4%	24.1%	42.8%	26.5%
400	800	400	3	46.5%	48.3%	49.8%	47.4%	33.3%	45.2%	48.5%	47.5%
400	800	400	6	37.5%	34.4%	40.2%	37.2%	23.6%	22.1%	27.9%	20.7%
400	800	800	3	46.9%	48.5%	49.7%	48.1%	31%	30%	48.1%	31.5%
400	800	800	6	37.7%	36.1%	40%	36.2%	24.4%	23.6%	27.2%	29.1%

When data are available on both GEs and regulators, studies under other contexts [11, 30] have directly conducted their joint analysis and observed improvement (over analyzing GE only). Here we also briefly experiment with such an approach. Specifically, in Table 3, we also consider an approach that we call augmented K-means (Aug-K). This approach is based on the K-means and includes both GEs and CNAs in the clustering algorithm. Specifically, K-means is adopted to analyze the stacked data (*Y*,*X*). To compare with other approaches, only the cluster memberships of GEs are tracked in the final results. Simulation results in Table 3 suggests that, unlike in regression analysis [17], directly integrating GEs and regulators fails in clustering analysis. Specifically, it tends to cluster different data types together, as opposed to clustering connected GEs and CNAs together. It is noted that this approach seems very stable. This is also reasonable. It tends to cluster all GEs in one big cluster. Thus, even though the results are wrong, they are very stable.

#### Remarks

Beyond those described above, we have also experimented with a few other settings and observed similar satisfactory performance of the proposed approach. In the above simulation, we have set *K*=2. We have examined similar simulation settings with *K*=3,…,10 and made similar observations. We have compared with the most popular alternatives, whose stable and satisfactory performance has been well observed in the literature. It is of interest to conduct more extensive comparisons with other (less popular) approaches in future studies.

### Data analysis

TCGA (https://tcga-data.nci.nih.gov/docs/publications/tcga/?) is one of the most recent multidimensional studies and has collected multiple types of omics measurements on the same subjects for multiple cancer types. The data have been recently collected and published and have a high quality. We analyze data on cutaneous melanoma (SKCM), which poses a serious public health concern. In this study, we analyze the processed level 3 data, which are downloaded from TCGA Provisional using the R package *CGDS*. We focus on metastatic samples of the Whites. Detailed information on data collection and processing is available on the TCGA website and elsewhere. Briefly, GE data were collected using the IlluminaHiseq RNAseq V2 platform and have been lowess-normalized, log-transformed, and median-centered. The robust Z-scores represent the gene expression status (up or down regulated) in tumor samples relative to normal tissues. A total of 19,626 measurements are available on 371 samples. CNA measurements were obtained using the Affymetrix Genome-wide Human SNP array 6.0 platform. The loss and gain levels of copy number changes of tumors compared to normal tissues are identified using segmentation analysis and expressed in the log2 transformed form. A total of 21,699 measurements are available on 366 samples. GE and CNA data are merged using sample ID, resulting in a total of 366 samples. There are a few missing measurements, which are filled in using imputation. Performance of the proposed approach (and alternatives) decays when the ratio of the number of unknown parameters and sample size increases. We first conduct a simple prescreening via marginal analysis based on overall survival (an important clinical outcome) and select the top 1000 most significant genes. We then use GOTerm Finder [31, 32] and search for the GO (Gene Ontology) biological processes. 382 out of the 1000 genes have well defined GO terms and are selected for downstream analysis.

Unlike in simulation, the number of clusters is unknown. Determining the optimal number of clusters is a challenging problem. In our data analysis, we adopt the GAP approach [33], which has been coupled with multiple clustering methods and extensively adopted in data analysis. With the GAP approach, *K*=4 clusters are constructed with the 382 GEs. For K-means, for comparability, we also set *K*=4. The spectral clustering is not considered because of its inferior performance in simulation. With ANCut, the cluster sizes are 110, 105, 97, and 70, respectively. With K-means, the cluster sizes are 27, 350, 1, and 4, respectively. The K-means results are less desirable with the dominating majority of genes in a single cluster. The other alternatives also have similar unsatisfactory results. Detailed clustering results are available from the authors.

**Fig. 3:**
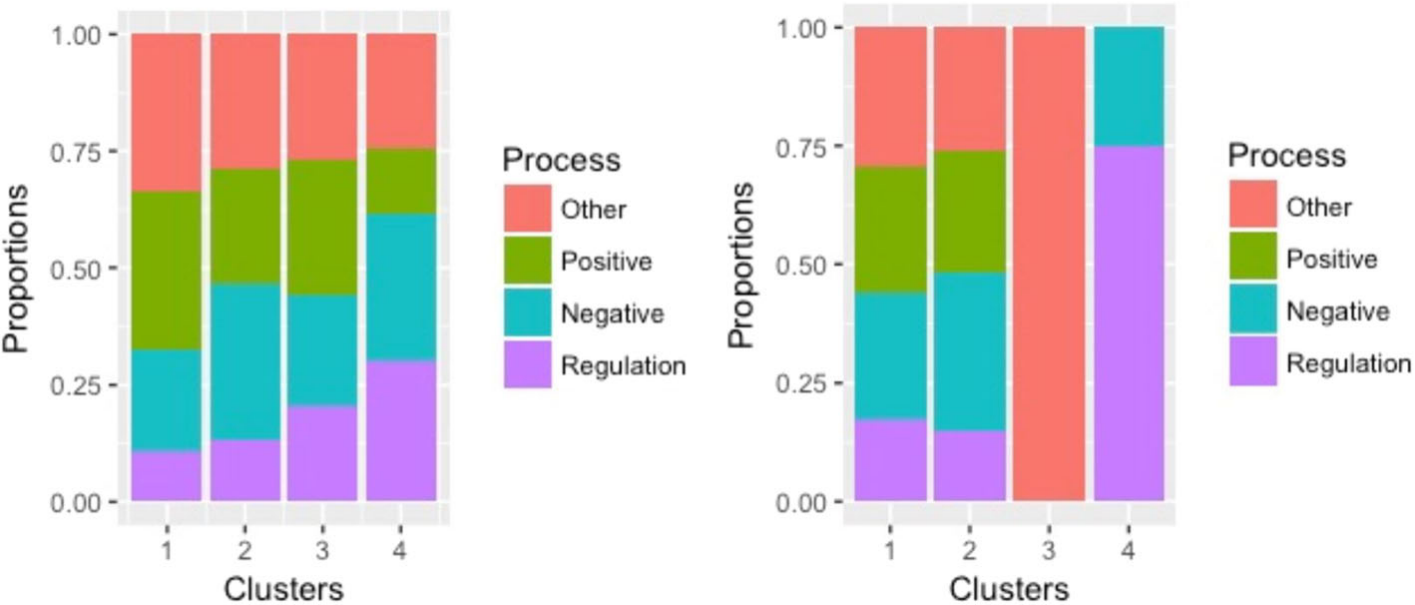
Functional modes. Analysis of TCGA data using ANCut (*left*) and K-means (*right*): the functional modes of the clusters

**Fig. 4:**
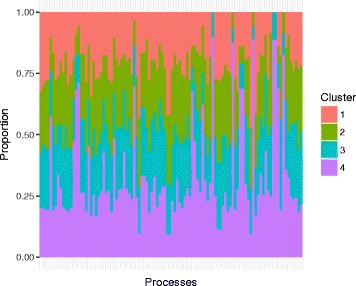
All GO processes. Analysis of TCGA data using ANCut: proportions of genes with a certain GO process in the four clusters

**Fig. 5:**
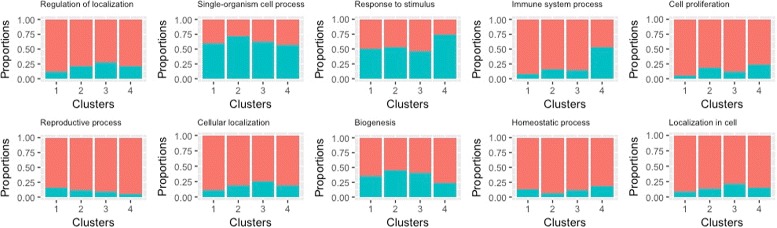
Selected GO processes. Analysis of TCGA data using ANCut: proportions of genes with a certain GO process in the four clusters

The 382 genes have a total of 104 GO biological processes. More closely examining these processes suggests that the majority are related to “regulation”. We further separate the 104 processes into four categories: positive regulation, negative regulation, regulation (without a well defined “direction”), and other. In Fig. 3, we compute the proportions of genes in the four clusters that have the four categories of processes. For ANCut, differences across the four clusters are clearly seen. For example, cluster 4 has a higher percentage of “regulation”, whereas cluster 1 has a higher percentage of “other”. With the highly unbalanced clustering, the K-means results, also presented in Fig. 3, are less interesting. For all of the 104 processes, we present their distributions in the four clusters in Fig. 4. Each bar represents one process, and different colors in the bar denote different proportions of genes in the clusters. Due to space limitation, the processes’ names are not shown. In general, the distributions of processes are quite different across clusters, which suggests the effectiveness of the proposed clustering. We further examine the ten most representative processes in detail. The results are presented in Fig. 5. It is shown that, for example, cluster 4 has a much higher percentage of “immune system process” than the other three clusters, while cluster 3 has a higher percentage of “localization in cell” than the others. Further examining the genes suggests that those annotated to the same processes are highly likely to be clustered together. For example, 90%, 88%, and 88% of the genes annotated to the processes “response to type I interferon”, “type I interferon signaling pathway”, and “cellular response to type I interferon” belong to cluster 4. With K-means, as the majority of genes are in a single cluster, such analysis is not conducted. The sensible biological findings provide support to the validity of the proposed clustering. Another finding from data analysis is that the results (distributions of biological processes) do depend on the number of clusters. The results with *K*=3 are presented in Figure A1 (Additional file 1). This finding is reasonable and has also been observed with other clustering methods in the literature.

## Discussion

Clustering is an important step of gene expression data analysis. Beyond having independent value, it has also served as the foundation of many other analyses. Although a large number of methods have been developed in the literature, since the practical results are often unsatisfactory, there is still a great demand for more effective methods. In the analysis of genetic data, a major problem is the “lack of information”. To tackle this problem, we have proposed conducting assisted analysis by borrowing information from the regulators of GEs. The proposed method is built on the NCut technique, which has multiple notable advantages but still limited applications in genetic data analysis. The proposed ANCut has been partly motivated by the recent “decomposing gene expressions” strategy and is the first to do so in clustering analysis. It has an intuitive formulation and can be effectively realized using an SA algorithm. In simulation, it outperforms the direct competitors with better accuracy and stability. We acknowledge that there are many other possible simulation settings and potentially applicable alternatives. The considered simulation settings have comprehensively covered multiple values of dimensionality, sample size, correlation, regulation strength, and others. The adopted alternatives are possibly the most popular in data analysis and thus warrant direct comparison. In the analysis of TCGA data, the ANCut analysis results are more desirable. It is noted that the SA algorithm encourages but not forces clusters with comparable sizes. Examining the GO terms suggests that the clustering results can be biologically sensible.

This study inevitably has multiple limitations, including a lack of theoretical investigation, still limited simulations, and a lack of functional analysis of the data analysis results. More extensive analysis, numerical studies and comparisons will be conducted in the future. We note that the strategy of using regulator information to assist GE clustering is not limited to the NCut technique. In Additional file 1, we briefly discuss the possibility of trying such a strategy with K-means. We focus on the ANCut in this study and defer systematic development of the assisted clustering analysis to future research.

## Conclusions

In this study, a new assisted analysis strategy is developed for clustering gene expression data. Advancing from the existing methods that focus on gene expression data only, information of regulators is utilized in clustering gene expressions. The proposed method carries a wealth of information and can reveal more accurate clustering. Experiment results on simulation and two TCGA datasets show the competitive performance of the proposed method with respect to the alternatives. This study provides a new venue for analyzing gene expression data. It is noted that although our analysis focuses on clustering of GEs, the proposed method is potentially applicable to other types of genetic measurements, such as proteins with their regulators.

## Additional file

Additional file 1 This file contains additional discussions and numerical results. (PDF 122 kb)

## Abbreviations

ANCut: Assisted normalized cut
Aug-K: Augmented K-means
CNA: Copy number alteration
GE: Gene expression
GO: Gene ontology
NCut: Normalized cut
SA: Simulated annealing
Spec: Spectral clustering
SKCM: Skin cutaneous melanoma
TCGA: The cancer genome atlas
