# Supplementary material for: Assisted clustering of gene expression data using ANCut
Source: BMC Genomics. 2017 Aug 16;18:623. doi: 10.1186/s12864-017-3990-1 (PMC5559859; doi:10.1186/s12864-017-3990-1)
Supplement: Additional file 1 — This file contains additional discussions and numerical results. (PDF 122 kb) [file 12864_2017_3990_MOESM1_ESM.pdf]

Additional File 1

Additional discussions on the NCut technique

As discussed in the main text, the NCut technique may have multiple much desired advantages. In addition, many clustering techniques can be viewed as special cases, extensions, or relaxations of NCut. For example, spectral clustering can be regarded as a relaxation of NCut, where the binary solution (in the case of $K = 2$) is relaxed to take continuous values (Von Luxburg, 2007). Compared to the relaxation versions, NCut has been shown to have additional advantages. Multiple examples are presented in Guattery and Miller (1998) and in Figure 2 of Von Luxburg (2007). In addition, both NCut and K-means can be expressed as a trace maximization problem. Thus K-means can be written as NCut as well (Dhillon et al., 2004). NCut may also have a sound statistical basis. It has been shown that the normalized spectral clustering, which is a relaxation of NCut, is consistent under very mild conditions. In contrast, some other graph cutting methods and their relaxations are only consistent under stringent conditions (Von et al., 2008). Another advantage of NCut is that it can be easily modified to serve the purpose of assisted analysis. Its numerator and denominator are "separated", making it easier to accommodate regulator information.

Additional data analysis results

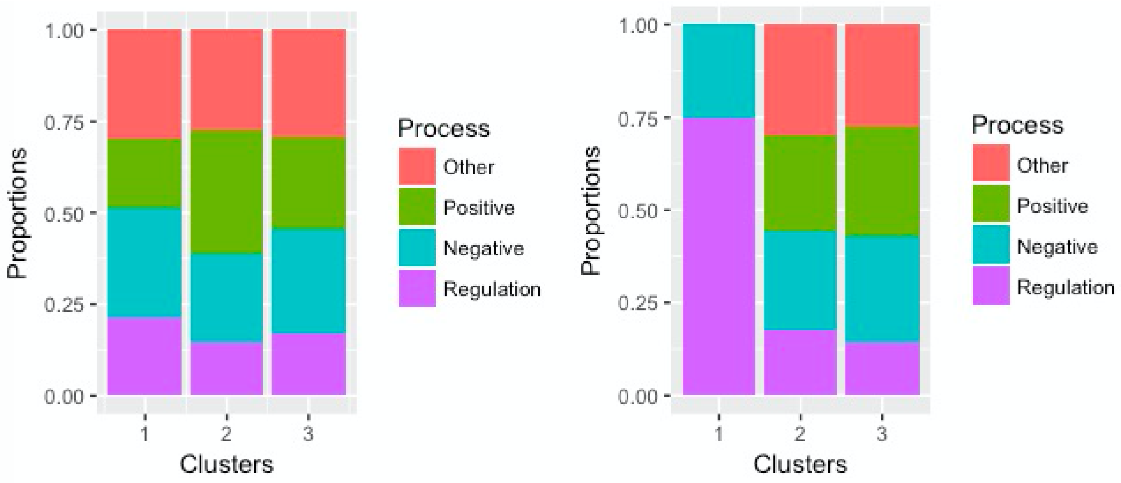

Figure A1: Functional modes: analysis of TCGA data using ANCut (left) and K-means (right) with K=3.

Assisted clustering based on other techniques

We note that the strategy of assisted clustering is not limited to the NCut technique. Below we briefly discuss the possibility of assisted clustering based on other techniques. We note that a systematic development of the assisted clustering is beyond the scope of this article and postponed to future research.

K-means For a given partition $A_1, \ldots, A_K$, the standard K-means objective function is

$$J(A_1, \ldots, A_K) = \sum_{k=1}^{K} \sum_{i \in A_k} ||\boldsymbol{Y}_i - \boldsymbol{m}_k||^2 \tag{1}$$

where $\boldsymbol{m}_k = \sum_{i \in A_k} \boldsymbol{Y}_i / n_k$, $|| \cdot ||$ is the Euclidean distance, and $n_k = |A_k|$ is the number of elements in A_k.

Following a similar spirit as for the ANCut, we may consider incorporating regulator information and modifying the K-means objective function as

$$AJ(A_1, \ldots, A_K) = \sum_{k=1}^{K} \sum_{i \in A_k} (1 - K(\boldsymbol{Y}_i, \boldsymbol{m}_k)) / \sum_{k=1}^{K} \sum_{i \in A_k} K(\hat{\boldsymbol{Y}}_i, \boldsymbol{m}_k) \tag{2}$$

where $K(\boldsymbol{Y}_i, \boldsymbol{m}_k) = exp(-||\boldsymbol{Y}_i - \boldsymbol{m}_k||/2\sigma^2)$ is the Gaussian kernel and $0 \leq K(\cdot, \cdot) \leq 1$. Here the numerator represents how dissimilar the data points $\boldsymbol{Y}_i$'s and class means $\boldsymbol{m}_k$'s are, and the denominator represents how similar $\hat{\boldsymbol{Y}}_i$'s are to $\boldsymbol{m}_k$'s.

Spectral clustering Consider the normalized spectral clustering algorithm in Shi and Malik (2000). This approach first finds the eigenvectors of the normalized Laplacian and then uses them for clustering data. The first eigenvector u_1 is the one that maximizes the following objective function

$$\frac{\boldsymbol{u}_1^T \tilde{\boldsymbol{L}} \boldsymbol{u}_1}{\boldsymbol{u}_1^T \boldsymbol{u}_1} \tag{3}$$

where $\tilde{\boldsymbol{L}} = \boldsymbol{D}^{-1}\boldsymbol{L}$, $\boldsymbol{D} = diag\{\sum_j w_{1,j}, \ldots, \sum_j w_{p,j}\}$ and $\boldsymbol{L} = \boldsymbol{D} - \boldsymbol{W}$ is the graph Laplacian. Other eigenvectors are derived similarly with the additional constraint of orthogonality. The first K eigenvectors are used to define K clusters. To accommodate regulator information, we may

consider modifying the objective function in (3) as

$$\frac{\boldsymbol{u}_1^T \tilde{\boldsymbol{L}} \boldsymbol{u}_1}{\boldsymbol{u}_1^T \bar{\boldsymbol{L}} \boldsymbol{u}_1} \tag{4}$$

where $\bar{\boldsymbol{L}} = \hat{\boldsymbol{D}}^{-1}\hat{\boldsymbol{L}}$, $\hat{\boldsymbol{D}} = diag\{\sum_j (1 - \hat{w}_{1,j}), \ldots, \sum_j (1 - \hat{w}_{p,j})\}$, and $\hat{\boldsymbol{L}} = \hat{\boldsymbol{D}} - (\boldsymbol{1} - \hat{\boldsymbol{W}})$ with $\boldsymbol{1}$ be the $p \times p$ matrix of ones. For the construction of both $\boldsymbol{W}$ and $\hat{\boldsymbol{W}}$, the Gaussian kernel is adopted so that it is guaranteed $0 \leq w_{i,j} \leq 1$ and $0 \leq \hat{w}_{i,j} \leq 1$. Similar to the objective function in (2), the eigenvector is found so that it maximizes the similarity among $\boldsymbol{Y}$'s and minimizes the dissimilarity among $\hat{\boldsymbol{Y}}$'s.

References

Dhillon, I. S., Guan, Y., and Kulis, B. (2004). K-means: spectral clustering and normalized cuts. *In Proceedings of the tenth ACM SIGKDD international conference on Knowledge discovery and data mining*, 551-556.

Guattery, S., and Miller, G. L. (1998). K-means: spectral clustering and normalized cuts. *SIAM Journal on Matrix Analysis and Applications*, 19(3), 701-719.

Shi, J., and Malik, J. (2000). Normalized cuts and image segmentation. *IEEE Transactions on Pattern Analysis and Machine Intelligence*, 22(8), 888-905.

Von Luxburg, U. (2007). A tutorial on spectral clustering. *Statistics and Computing*, 17(4), 395-416.

Von Luxburg, U., Belkin, M., and Bousquet, O. (2008). Consistency of spectral clustering. *The Annals of Statistics*, 36(2), 555-586.
